# How to bring generative AI to oncology practice

**DOI:** 10.1016/j.esmorw.2025.100679

**Published:** 2026-01-30

**Authors:** D. Truhn, J.N. Kather

**Affiliations:** 1 Diagnostic and Interventional Radiology, University Hospital Aachen, Aachen, Germany; 2 Lab for AI in Medicine, University Hospital Aachen, Aachen, Germany; 3 Else Kroener Fresenius Center for Digital Health, Faculty of Medicine, TUD Dresden University of Technology, Dresden, Germany; 4 Department of Medicine I, Faculty of Medicine, TUD Dresden University of Technology, Dresden, Germany; 5 Medical Oncology, National Center for Tumor Diseases (NCT), University Hospital Heidelberg, Heidelberg, Germany

**Keywords:** artificial intelligence, oncology, large language models, generative AI

## Abstract

Generative artificial intelligence (AI) is entering oncology. Large language models are the near-term workhorse because oncology runs on narrative text and structured tables. We review current adoption and outline a practical path from stand-alone chat models to retrieval-augmented systems and, ultimately, agentic assistants that plan tasks, call domain tools, and integrate multimodal data within the electronic health record. Concrete uses include molecular tumor board synthesis with transparent evidence, grading along guidelines, synoptic radiology and pathology drafting, and computable trial matching. We also map the constraints: fragmented hospital information technology, privacy and provenance requirements, domain shift across sites, and persistent hallucinations. We envision that evaluation must move beyond leaderboards toward multicenter, prospective designs with endpoints that reflect clinical utility, such as faithfulness to cited sources, extraction accuracy, time to task completion, plan correctness, recovery after tool failure, and silent clinical studies before exposure. Finally, we sketch an adoption trajectory. Institutions will replace *ad hoc* use of public tools with sanctioned drafting assistants, then embed retrieval and calculators inside the record, and only later enable event-driven agents that propose context-aware actions. The destination is augmentation, not automation: a learning assistant that shows its work, improves routine care, and leaves clinical judgment with clinicians.

## Highlights

• Generative AI can simplify oncology workflows and reduce routine documentation work.
• Large language models can reason over narrative and structured medical data.
• Adoption will be gradual, from drafting tools to integrated clinical assistants.
• Meaningful use requires transparency, validation, and human oversight.

## Introduction

Traditionally, deep learning models have been used for classification. For example, when provided with an image, the deep learning model classifies the image into different baskets, for example ‘cancer’, or ‘no cancer’. Generative artificial intelligence (AI), on the other hand, refers to models that generate new data. These data can be images, video, sound, or text. The latter is generated by the most prominent class of generative AI models: large language models (LLMs). Provided with an input (the user’s text), the LLM generates new text-data, namely the model’s response.^1^

For oncology, the most practical generative AI models are LLMs. The field runs on text and structured tables: guidelines, protocols, molecular reports, pathology and radiology narratives, clinic notes, trial registries, and patient messages. An assistant that can read across these sources, keep track of entities and time, draft with citations, and expose its evidence fits the daily workflow of clinics and tumor boards. Image or video generative models do not have broad clinical applicability yet. Therefore, this article will concentrate on text-generative models, i.e. LLMs.

At their core, LLMs are trained to predict the next word in a sequence. Consequently, they function as sophisticated sentence continuation engines that must understand text content to accurately forecast what comes next. For example, given the context ‘the patient presents with untreated prostate carcinoma, the PSA level is ___’, the model learns to predict ‘elevated’ with a higher probability than ‘low’. By solving these statistical puzzles at scale, the LLM incorporates medical reasoning and pattern recognition into its internal representations.^2^^,^^3^ Many clinical processes follow a similar logic: in molecular tumor boards, a patient’s specific biomarker constellation is pattern-matched to guidelines and standard operating procedures. When an LLM encounters ‘metastatic colorectal cancer’ combined with a ‘BRAF V600E mutation’, it assigns a higher probability to a guideline-concordant continuation involving a BRAF inhibitor rather than standard chemotherapy alone. In this view, the correct therapeutic decision becomes simply the logical, semantic completion of the patient’s clinical narrative.

The remainder of this article outlines a practical roadmap for bringing generative AI to the clinic. We begin by categorizing LLMs into levels of functional complexity, which serves as a basis for understanding their potential applications in oncology. Finally, we address the critical obstacles and suggest a path for validation and adoption.

## Levels of complexity in language model applications

**Figure 1:**
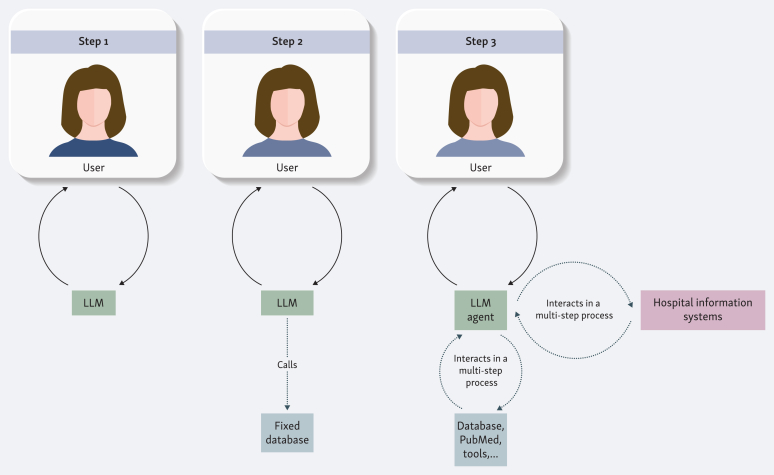
**Levels of complexity in language model applications for oncology practice.** Step 1 shows a stand-alone large language model (LLM) that generates responses solely from its pre-trained parameters. Step 2 illustrates retrieval-augmented generation, where the LLM calls a fixed, curated database to provide more up-to-date and explainable outputs. Step 3 represents agentic AI: an LLM agent that interacts in multistep processes with multiple external tools, databases, and hospital information systems, enabling integrated decision support and workflow assistance.

It is useful to think of LLM assistants in terms of different levels of complexity (see Figure 1). At the simplest level stands the self-contained language model, exemplified by the early chat systems such as the first version of ChatGPT. Such a model accepts a query and generates an answer entirely from the knowledge embedded in its parameters at the close of training. An oncologist might, for instance, ask about the adverse event profile of a chemotherapy regimen or the necessary dose adjustments in renal impairment and receive a coherent summary that lists typical toxicities and outlines monitoring strategies. The limitation, however, is fundamental. Once training is complete, no new information can be integrated. Emerging trial results, label modifications, and updated guidelines remain outside the reach of the model, no matter how fluent the response. A further risk is that when the model lacks relevant knowledge, it may none the less generate a confident but incorrect statement. This is a phenomenon now widely referred to as hallucination.^4^

The usefulness of such stand-alone models for clinical reality is quickly exhausted. The next step in complexity is represented by language models that, although fixed in their training parameters, are given the ability to query external databases. This approach is known as retrieval-augmented generation. Instead of relying solely on internalized knowledge, the model is provided with access to curated sources that can be searched by the LLM at the time of the query. In oncology, such a source might be a continually updated repository of anticancer drugs with their adverse event profiles and dosing recommendations. Thus it functions less as a static oracle and more as a dynamic and explainable search engine tailored to clinical needs.^5^

The third step is agentic AI, which many experts see as the most plausible entry point of advanced AI into routine oncology practice. In contrast to static models or retrieval-augmented systems, an agentic model not only produces text but also reasons about the data and acts on its environment. It can plan a sequence of steps, invoke external tools, evaluate their outputs, and revise its strategy until it arrives at a coherent answer. This active role transforms the model into an assistant that can connect disparate data sources and produce integrated outputs rather than isolated fragments. In oncology, this may mean invoking a pathology model that predicts microsatellite instability or KRAS and BRAF status directly from routine histology, using a radiology segmentation tool to quantify lesion growth, calculating creatinine clearance to adjust dosing, or retrieving evidence from PubMed and OncoKB before proposing a therapy recommendation. Early evaluations suggest that such agents can improve decision accuracy and assist in clinical routine.^6^

## Clinical use cases and challenges

The clinical possibilities are broad: for molecular tumor boards, an agent could assemble variant information, check mutations against established international oncology frameworks and guidelines, and summarize therapeutic and trial options with explicit eligibility matches. In documentation, it could turn audio transcripts into structured notes, classify side-effects according to standard severity grading systems, and draft synoptic pathology or radiology reports structured according to widely used pathology and radiology reporting standards. For clinical trials, it could translate free-text eligibility criteria into structured, searchable rules, scan patient registries, and generate transparent rationales for inclusion or exclusion.^7^, ^8^, ^9^ At first, such agents will likely support routine, low-risk tasks such as drafting notes or assembling background information for tumor boards. Over time, with careful validation, they may extend into complex decision support, acting as a colleague that synthesizes multimodal data and literature to propose, not impose, individualized treatment strategies.

### Barriers to deployment

However, real deployment faces practical obstacles: most hospital software systems are fragmented and data access is guarded with privacy and safety in mind. Most share data only through narrow, sometimes fragile interfaces, and access is tightly controlled with user roles and full audit logs. Any assistant that reads from the chart and writes back must stay within those guardrails. It also requires a secure, local repository of the texts and guidelines the assistant searches, and clear version control for the models so that changes can be tracked and reversed. Anything the system adds to the record should carry a label that shows how it was produced: the question asked, the sources consulted, the tools it invoked, the model version, and an estimate of confidence. Without this level of transparency, fixing errors becomes guesswork and responsibility becomes unclear.

### Robustness and evaluation

Robustness across settings is the second hard problem. Models tuned on one hospital’s data drift when confronted with another hospital’s note style, laboratory panels, staining protocols, or scanner vendors. Domain shift degrades extraction accuracy and silently changes recommendations.^10^ The only remedy is prospective, multicenter evaluation with pre-registered endpoints and reporting by subgroup. External validation should include adversarial tests, long documents, code-mixed text, and counterfactual prompts. Agents need abstention behavior when context is missing or out of distribution, along with clear escalation to a human reviewer. Tool wrappers should enforce schema checks and unit bounds so that a malformed input to a calculator, or a mislabeled image slice, does not cascade into a wrong plan. To determine whether these safeguards hold in real clinical use, evaluation must move beyond the leaderboard culture that ranks models on narrow, single-task benchmarks that are often not reflective of clinical reality. Clinically relevant endpoints should include faithfulness to cited evidence, time to task completion, error rates on structured extraction, and net benefit using decision-curve analysis. For agents, measuring plan quality, the correctness of each tool call, recovery after tool failure, and stability across repeated runs are important measures. A useful intermediate step is the evaluation in a so-called silent clinical study: let the agent produce an answer that is never shown to the clinician, then compare against the ground truth from tumor board minutes or signed reports.

### Regulatory, technical, and human factors

Only when performance is stable across sites should the system graduate to a mode where its outputs are used for clinical decision making, and even then all outputs should remain editable and attributable. This naturally leads to the legal and regulatory dimension, which cannot be treated as a footnote. If an agent only retrieves passages and drafts narrative text for a human to sign, it is assistive documentation. The moment it writes discrete data into the electronic record or proposes executable orders, it looks like a medical device. That brings quality management systems, post-market surveillance, change control, and sometimes pre-market review under national and regional frameworks. The EU AI Act will likely classify such systems as high-risk.^11^ In the United States, Software as a Medical Device principles apply when outputs influence diagnosis or therapy. Institutions will need model update policies that resemble drug formulary management. Version changes cannot be silent. They require regression testing and a traceable approval path. Vendors and hospitals also need data-processing agreements that cover tool calls to external services, since some tools may leave the firewall by design.^12^ Beyond these legal and organizational safeguards, there remain technical limitations that affect reliability in daily use. Chief among them are hallucinations, which persist even when retrieval mechanisms are in place.^13^ A grounded system reduces fabrication but does not eliminate it. Models can misattribute a sentence, paraphrase beyond the scope of the source, or may confuse similar drug names and indications. Tool outputs can be misread. The safe pattern is evidence before conclusion. Show the passages and highlight the part of the guideline that a decision is based on, and present calculations with inputs and units. If the evidence is thin or contradictory, the agent should say so and ask for clarification rather than fill the gap with plausible prose. This is a problem that still needs to be solved as most paradigms for training of these systems still do not reward abstention, but rather trial and error.^14^

But even the best-calibrated system cannot replace human judgment. The reliability of these tools ultimately depends on how they are used, which brings us to the human side of the equation: people matter as much as models. Clinicians should have the opportunity to build familiarity with such systems, either through everyday practice, structured exposure, or optional courses. Developing this familiarity means cultivating habits that balance efficiency with vigilance: formulating clear prompts, verifying the evidence behind summaries, checking dates and lines of therapy, and deciding when to escalate. Interface design can support this process by placing citations and tool traces ahead of recommendations, requiring an extra confirmation for high-risk actions, and logging any human edits. Such habits are essential, because the literature already documents automation bias and anchoring when AI presents confident suggestions.^15^ Without conscious checks, clinicians may over-rely on the system, leading to error propagation and, over time, to deskilling if left unchecked.

These risks highlight that human oversight is important, but oversight alone is not enough. For agents to become genuinely reliable, they also need to be trained on the right foundations. Data remain the limiting reagent. An agent can only learn oncology if we teach it with oncology. That requires consented, well-curated corpora that cover the modalities we actually use: notes, structured tables, radiology images, whole-slide pathology images, genomic reports, and longitudinal outcomes. As the datasets are massive, weak supervision and self-supervised pretraining will probably be needed to reach scale. Nevertheless, expert-generated labels will at least be needed to generate reliable and challenging test cases for these systems. And for fine-tuning, the most valuable labels live in routine edits. Every strike-through, every added sentence, every corrected grade is a supervision signal. With governance and explicit consent, those edits can feed continuous improvement rather than disappear into the ether. Table 1 summarizes the challenges and corresponding requirements for deploying agentic AI systems that plan tasks, call domain tools, and integrate multimodal data.

**Table 1:** Key considerations for deployment of generative AI in oncology practice

Key challenge	Requirements for generative AI success
IT Infrastructure and transparency: fragmented hospital systems with narrow interfaces limit data access.	Interoperability: hospital interfaces must be interoperable and accessible to AI systems to ensure seamless data flow.
Robustness across settings: domain shift due to varying note styles, laboratory panels, or SOPs across sites degrades performance.	Multicenter validation: generative AI must be validated on real-world multicenter data (also see below). Stress testing: protocols must include adversarial tests, long documents, code-mixed text, and counterfactual prompts.
Real-world evaluation: standard ‘leaderboard’ benchmarks do not reflect clinical reality.	Clinical benchmarks: evaluation must use real-world data and reflect tasks typically undertaken by clinicians. Silent studies: agents should run in ‘silent mode’ (shadow mode), where outputs are compared with ground truth but not shown to clinicians before live exposure.
Hallucinations: LLMs are prone to hallucinating or fabricating information.	Abstention protocols: agents must identify missing context and escalate to humans rather than guessing. ‘Evidence-first’ UI: the interface must enforce ‘evidence before conclusion’, displaying cited passages and calculations before the recommendation.
Regulatory and governance: Legal frameworks lag behind the pace of technological development.	Clear pathways: feasible, clear pathways for regulatory compliance must be established to facilitate the safe introduction of generative AI into clinics.
Human factor: risks of automation bias and the deskilling of junior physicians.	Engagement and education: AI must be integrated in a way that maintains human engagement/oversight, ensuring that young physicians continue to learn and retain core skills.
Data foundations: lack of specific, curated oncology training data.	Multimodal datasets: large-scale, multimodal oncology datasets must be made available to train models.

AI, artificial intelligence; IT, information technology; LLMs, large language models; SOPs, standard operating procedures; UI, user interface.

## Path to adoption

A pragmatic adoption path is already visible. The first step is happening in the shadows. Many clinicians use general chat systems to draft letters or reorganize notes. The behavior reflects real pain points. It also risks privacy breaches and inconsistent quality. Institutions will probably meet this reality by providing sanctioned, private alternatives that forbid entry of protected health information into public tools. The second step is the arrival of dedicated assistants inside the electronic health record. These systems will see the patient context through the hospital information system, retrieve approved content, and draft notes and patient letters with citations. For the foreseeable future, they will not act without human approval. Tooling will be modest at first. Think of tools that grade side-effects, calculate safe drug doses, and match patients to clinical trials using structured data. Even this level can save hours each week and raise documentation quality. The third step is deep integration and broader context. Agents become event driven. A new pathology result triggers a proposed tumor board summary. A rising creatinine triggers a suggested dose adjustment with evidence links. A new trial at the institution prompts a list of likely candidates with eligibility logic attached. Multimodal capability matures. The system reads radiology and pathology alongside notes and labs, calls specialist models as needed, and renders both narrative and computable output. All of this only works if we pursue data and evaluation in parallel. The community needs to invest in shared benchmarks that look like real work. Examples include blinded reviews of tumor board summaries, measuring how precisely and completely patients are matched to trials, checking the accuracy of side-effect grading from raw notes, and assessing how faithfully structured reports are generated. These should be multicenter by design and reported with subgroup analyses. Negative results need to see daylight. At the same time, curated training sets that reflect the diversity of patients and practice need to be set up. Small centers and community clinics must be part of the loop or the technology will amplify existing inequities.^16^

The destination is not a machine that replaces clinicians. It is a learning system that helps them see the right evidence at the right moment and records exactly how it reached a suggestion. We are at the early stage where the value comes from better retrieval and better drafts. With careful engineering, rigorous evaluation, and a culture of human oversight, agents will move from drafting to real decision support.
